# Psychosocial treatment and interventions for bipolar disorder: a systematic review

**DOI:** 10.1186/s12991-015-0057-z

**Published:** 2015-07-07

**Authors:** Stella Miziou, Eirini Tsitsipa, Stefania Moysidou, Vangelis Karavelas, Dimos Dimelis, Vagia Polyzoidou, Konstantinos N Fountoulakis

**Affiliations:** Aristotle University of Thessaloniki, Thessaloníki, Greece; Division of Neurosciences, 3rd Department of Psychiatry, School of Medicine, Aristotle University of Thessaloniki, 6, Odysseos Street (1st Parodos, Ampelonon Str.), Pournari Pylaia, 55535 Thessaloníki, Greece; Thessaloníki, Greece

## Abstract

**Background:**

Bipolar disorder (BD) is a chronic disorder with a high relapse rate, significant general disability and burden and with a psychosocial impairment that often persists despite pharmacotherapy. This indicates the need for effective and affordable adjunctive psychosocial interventions, tailored to the individual patient. Several psychotherapeutic techniques have tried to fill this gap, but which intervention is suitable for each patient remains unknown and it depends on the phase of the illness.

**Methods:**

The papers were located with searches in PubMed/MEDLINE through May 1st 2015 with a combination of key words. The review followed the recommendations of the Preferred Items for Reporting of Systematic Reviews and Meta-Analyses statement.

**Results:**

The search returned 7,332 papers; after the deletion of duplicates, 6,124 remained and eventually 78 were included for the analysis. The literature supports the usefulness only of psychoeducation for the relapse prevention of mood episodes and only in a selected subgroup of patients at an early stage of the disease who have very good, if not complete remission, of the acute episode. Cognitive-behavioural therapy and interpersonal and social rhythms therapy could have some beneficial effect during the acute phase, but more data are needed. Mindfulness interventions could only decrease anxiety, while interventions to improve neurocognition seem to be rather ineffective. Family intervention seems to have benefits mainly for caregivers, but it is uncertain whether they have an effect on patient outcomes.

**Conclusion:**

The current review suggests that the literature supports the usefulness only of specific psychosocial interventions targeting specific aspects of BD in selected subgroups of patients.

## Background

Our contemporary understanding of bipolar disorder (BD) suggests that there is an unfavorable outcome in a significant proportion of patients [1, 2]. In spite of recent advances in pharmacological treatment, many BD patients will eventually develop chronicity with significant general disability and burden. The burden will be significant also for their families and the society as a whole [3, 4]. Today, we also know that unfortunately, symptomatic remission is not identical and does not imply functional recovery [5–7].

Since pharmacological treatment often fails to address all the patients’ needs, there is a growing need for the development and implementation of effective and affordable interventions, tailored to the individual patient [8]. The early successful treatment, with full recovery if possible, as well as the management of subsyndromal symptoms and of psychosocial stress and poor adherence are factors predicting earlier relapse and poor overall outcome [9, 10].

In this frame, there are several specific adjunctive psychotherapies which have been developed with the aim of filling the above gaps and eventually improve the illness outcome [11], but it is still unclear whether they truly work and which patients are eligible and when [12–19].

The current study is a systematic review of the efficacy of available psychosocial interventions for the treatment of adult patients with BD.

## Methods

Reports investigating psychotherapy and psychosocial interventions in BD patient samples were located with searches in Pubmed/MEDLINE through May 1, 2015. Only reports in English language were included.

The Pubmed database was searched using the search terms ‘bipolar’ and ‘psychotherapy’ or ‘cognitive-behavioral’ or ‘CBT’ or ‘psychoeducation’ or ‘interpersonal and social rhythm therapy’ or ‘IPSRT’ or ‘family intervention’ or ‘family therapy’ or ‘group therapy’ or ‘intensive psychosocial intervention’ or ‘cognitive remediation’ or ‘functional remediation’ or ‘Mindfulness’.

The following rules were applied for the selection of papers:

1. Papers in English language.
2. Randomized controlled trials.

This review followed the recommendations of the Preferred Items for Reporting of Systematic Reviews and Meta-Analyses (PRISMA) statement [20].

## Results

**Figure 1:**
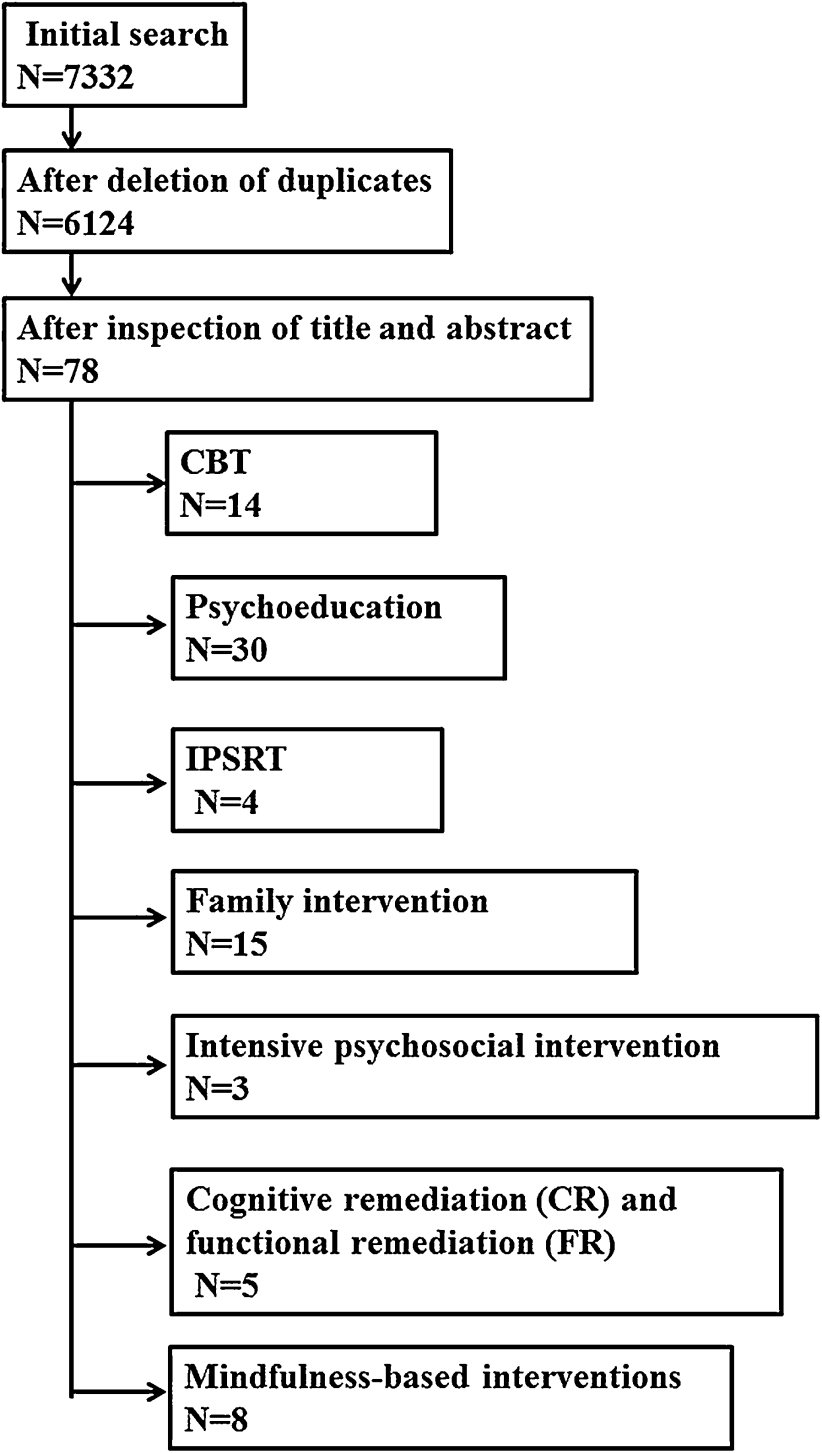
The PRISMA flowchart.

The search returned 7,332 papers, and after the deletion of duplicates 6,124 remained for further assessment. After assessing these papers on the basis of title and abstract, the remaining papers were (Figure 1). The number of paper reported for each intervention includes RCTs, post hoc analyses and meta-analyses together.

### Cognitive-behavioural therapy (CBT)

The efficacy of CBT in BD was investigated in 14 studies which utilized CBT as adjunct treatment to pharmacotherapy or treatment as usual (TAU). They utilized some kind of control intervention which should not be considered as an adequate placebo. It is also interesting that the oldest study was conducted in 2003.

This first study lasted 12 months and concerned 103 BD-I patients during the acute depressive phase and randomized them to 14 sessions of CBT or a control intervention. There was not any placebo condition. These authors reported that at end point fewer patients in the CBT group relapsed in comparison to controls (44 vs. 75%; HR = 0.40, *P* = 0.004), had shorter episode duration, less admissions and mood symptoms, and higher social functioning [21]. It was disappointing that the extension of this study (18 months follow-up) was negative concerning the relapse rate [22].

A second trial included 52 BD patients and was also negative concerning the long-term efficacy after comparing CBT plus additional emotive techniques vs. TAU [23]. On the other hand, the comparison of CBT plus psychoeducation vs. TAU in 40 BD patients reported a beneficial effect even after 5 years in terms of symptoms and social–occupational functioning. However, that study did not report the rate of recurrences and the time to recurrence [24]. A study in 79 BD patients (52 BD-I and 27 BD-II) compared CBT plus psychoeducation vs. psychoeducation alone and reported that the combined treatment group had 50% fewer depressed days per month, while at the same time the psychoeducation alone group had more antidepressant use [25]. Another study on 41 BD patients randomized to CBT vs. TAU reported similar results and an improvement in symptoms, frequency and duration of episodes [26].

An 18-month study compared CBT vs. TAU in 253 BD patients and reported that at end point, there were no differences between groups with more than half of the patients having a recurrence. It is interesting that a post hoc analysis suggested that CBT was significantly more effective than TAU in those patients with fewer than 12 previous episodes, but less effective in those with more episodes [13]. Similar negative results were reported concerning the number of episodes and time to relapse by another 12-month study of CBT vs. TAU in 50 BD patients in remission [17]. Again, negative findings concerning the relapse rate were reported by a 2-year study on 76 BD patients randomized to receive 20 sessions of CBT vs. support therapy [15]. Finally, the use of combined CBT and pharmacotherapy in 40 patients with refractory bipolar disorder suggested that the combination group had less hospitalization events in comparison to the group in the 12-month evaluation (*P* = 0.015) and lower depression and anxiety in the 6-month (*P* = 0.006; *P* = 0.019), 12-month (*P* = 0.001; *P* < 0.001) and 5-year (*P* < 0.001, *P* < 0.001) evaluation time points. However it is interesting that after the 5-year follow-up, 88.9% of patients in the control group and 20% of patients in the combination group showed persistent affective symptoms and difficulties in social–occupational functioning [27].

The use of CBT in BD comorbid with social anxiety disorder is of doubtful efficacy [28], while there are some preliminary data on the efficacy of an Internet-based CBT intervention [29] as well as recovery-focused add-on CBT [30] and CBT for insomnia [31] in comparison to TAU.

The review of the available data so far give limited support for the usefulness of CBT during the acute phase of bipolar depression as adjunctive treatment in patients with BD, but definitely not for the maintenance phase. During the maintenance phase, booster sessions might be necessary, but the data are generally overall negative. Probably, patients at earlier stages of the illness might benefit more from CBT. Unfortunately the type of patients who are more likely to benefit from CBT constitutes a minority in usual clinical practice.

### Psychoeducation

The basic concept behind psychoeducation for BD concerns the training of patients regarding the overall awareness of the disorder, treatment adherence, avoiding of substance abuse and early detection of new episodes. The efficacy of psychoeducation in BD was investigated in 30 studies, all of which utilized psychoeducation as adjunct treatment to pharmacotherapy or TAU. All these studies utilize some kind of control intervention which should not be considered as an adequate placebo. It is also interesting that the oldest study was conducted in 1991.

The earliest psychoeducational study was open and uncontrolled and reported that giving information about lithium improved the overall attitude towards treatment [32, 33]. A similar small study was conducted a few years later and reported similar results [34]. However, the first study on the wide teaching of patients to recognize and identify the components of their disease with emphasis on early symptoms of relapse and recurrence and to seek professional help as early as possible had not been conducted until 1999. It included 69 patients for 18 months and compared psychoeducation (limited number of sessions; 7–12) vs. TAU. It reported a significant prolongation of the time to first manic relapse (*P* = 0.008) and significant reductions in the number of manic relapses over 18 months (30 vs. 52%; *P* = 0.013) as well as improved overall social functioning. Psychoeducation had no effect on depressive relapses [35].

In a more systematic way, the efficacy of the adjunctive group psychoeducation was tested by the Barcelona group. Their trial included 120 euthymic BD patients who were randomly assigned to 21 sessions of group psychoeducation vs. non-specific group meetings. The study included a follow-up with a duration of 2 and 5 years. The results suggested that psychoeducation exerted a beneficial effect on the rate of and the time to recurrence as well as concerning hospitalizations per patient. At the end of the 2-year follow-up, 23 subjects (92%) in the control group fulfilled the criteria for recurrence versus 15 patients (60%) in the psychoeducation group (*P* < 0.01). This beneficial effect was high and was not reduced after 5 years (any episode 0.79 vs. 0.87; mania 0.40 vs. 0.57; hypomania 0.27 vs. 0.42 and mixed episodes 0.34 vs. 0.61), except for depressive episodes (0.91 vs. 0.80) [36–38].

The literature suggests that psychoeducation should be broad and that enhanced relapse prevention alone does not seem to work. This was the conclusion from another study with a different design. That study reported that only occupational functioning, but not time to recurrence, improved with an intervention consisting of training community mental health teams to deliver enhanced relapse prevention [39]. Additionally, a study with a 12-month follow-up and with a similar design to the first study of the Barcelona group, but with 16 sessions, reported no differences between groups in mood symptoms, psychosocial functioning and quality of life. It did find, however, that there was a difference in the subjectively perceived overall clinical improvement by subjects who received psychoeducation. The authors suggested that characteristics of the sample could explain this discrepancy, as patients with a more advanced stage of disease might have a worse response to psychoeducation [16]. In accordance with the above, a post hoc analysis of the original Barcelona data revealed that patients with more than seven episodes did not show significant improvement with group psychoeducation in time to recurrence, and those with more than 14 episodes did not benefit from the treatment in terms of time spent ill [40]. A 2-year follow-up in 108 BD patients investigated psychoeducation plus pharmacotherapy vs. pharmacotherapy alone. Psychoeducation concerned eight, 50-min sessions of psychological education, followed by monthly telephone follow-up care and psychological support. The results suggested that psychoeducation improved medication compliance (*P* = 0.008) and quality of life (*P* < 0.001) and had fewer hospitalizations (*P* < 0.001) [41]. Another study randomized 80 BD patients to either the psychoeducation or the control group and reported that the psychoeducation group scored significantly higher on functioning levels (emotional functioning, intellectual functioning, feelings of stigmatization, social withdrawal, household relations, relations with friends, participating in social activities, daily activities and recreational activities, taking initiative and self-sufficiency, and occupation) (*P* < 0.05) compared with the control group after psychoeducation [42].

A prospective 5-year follow-up of 120 BD patients suggested that group psychoeducation might be more cost-effective [43]. In support of the cost-effectiveness of psychoeducation was one trial in 204 BD patients which compared 20 sessions of CBT vs. 6 sessions of group psychoeducation and reported that overall the outcome was similar in the two groups in terms of reduction of symptoms and likelihood of relapse, but psychoeducation was associated with a decrease of costs ($180 per subject vs. $1,200 per subject for CBT) [44] Currently, there are some proposals of online psychoeducation programmes, but results are still inconclusive or pending [45, 46].

More complex multimodal approaches and multicomponent care packages have been developed and usually psychoeducation is a core element. One of these packages also included CBT and elements of dialectical behaviour therapy and social rhythms and has shown a beneficial effect after the 1-year follow-up in comparison to TAU [47]. Another included a combination of CBT plus psychoeducation and reported that it was more effective in comparison to TAU in 40 refractory BD patients concerning hospitalization and residual symptoms at 12 months follow-up [27]. A collaborative care study on 138 patients and follow-up of 12 months also gave positive results [48]. One multicentred Italian study assessed the efficacy of the Falloon model of psychoeducational family intervention (PFI), originally developed for schizophrenia management and adapted to BD-I disorder. It included 137 recruited families, of which 70 were allocated to the experimental group and 67 to the TAU group. At the end of the intervention, significant improvements in patients’ social functioning and relatives’ burden were found in the treated group compared to TAU [49]. In general, the beneficial effect seems to be present concerning manic but not depressive episodes [50, 51], while a benefit on social role function and quality of life seems also to be present [50].

The comparison of 12 sessions of psychoeducation vs. TAU in 71 BD patients reported that at 6 weeks, the intervention improved treatment adherence [52], while another on 61 BD-II patients reported no significant effect on the regulation of biological rhythms when compared to standard pharmacological treatment [53]. No significant effect was reported concerning the quality of life by another recent study on 61 young bipolar adults [54]. On the contrary, a trial on 47 BD patients reported that a psychoeducation programme designed for internalized stigmatization may have positive effects on the internalized stigmatization levels of patients with bipolar disorder [55].

There is preliminary evidence that a Web-based treatment approach in BD (‘Living with Bipolar’—LWB intervention) is feasible and potentially effective [56]; however, other Web-based attempts returned negative results [57]. Automated mobile-phone intervention is another option and it has been reported to be feasible, acceptable and might enhance the impact of brief psychoeducation on depressive symptoms in BD. However, sustainment of gains from symptom self-management mobile interventions, once stopped, may be limited [58].

One meta-analysis of 16 studies, 8 of which provided data on relapse reported that psychoeducation appeared to be effective in preventing any relapse (OR: 1.98–2.75; NNT: 5–7) and manic/hypomanic relapse (OR: 1.68–2.52; NNT: 6–8), but not depressive relapse. That meta-analysis reported that group, but not individually, delivered interventions were effective against both poles of relapse [59].

In summary, the literature suggests that interventions of 6-month group psychoeducation seem to exert a long-lasting prophylactic effect. However this is rather restricted to manic episodes and to patients at the earlier stages of the disease who have achieved remission before the intervention has started. Although the mechanism of action of psychoeducation remains unknown, it is highly likely that the beneficial effect is mediated by the enhancement of treatment adherence, the promoting of lifestyle regularity and healthy habits and the teaching of early detection of prodromal signs.

### Interpersonal and social rhythm therapy (IPSRT)

Interpersonal and social rhythm therapy is based on the hypothesis that in vulnerable individuals, the experience of stressful life events and unstable or disrupted daily routines can lead to affective episodes via circadian rhythm instability [18]. In this frame, IPSRT includes the management of affective symptoms through improvement of adherence to medication and stabilizing social rhythms and the resolution of interpersonal problems. Four papers investigating its efficacy were identified.

The first study concerning its efficacy in BD included 175 acutely ill BD patients and followed them for 2 years. It included four treatment groups, reflecting IPSRT vs. intensive clinical management during the acute and the maintenance phase. The results revealed no difference between interventions in terms of time to remission and in the proportion of patients achieving remission (70 vs. 72%), although those patients who received IPSRT during the acute treatment phase survived longer without an episode and showed higher regularity of social rhythms [60]. In spite of some encouraging findings from post hoc analysis, there were eventually no significant differences between genders and concerning the improvement in occupational functioning [61]. More recently, a 12-week study in which unmedicated depressed BD-II patients were randomized to IPSRT (*N* = 14) vs. treatment with quetiapine (up to 300 mg/day; *N* = 11), showed that both groups experienced significant reduction in symptoms over time, but there were no group-by-time interactions. Response and drop-out rates were similar [62]. Finally, one 78-week trial investigated the efficacy of IPSRT vs. specialist supportive care on depressive and mania outcomes and social functioning, and mania outcomes in 100 young BD patients. The results revealed no significant difference between therapies [63].

Overall, there are no convincing data on the usefulness of IPSRT during the maintenance phase of BD. There are, however, some data suggesting that if applied early and particularly already during the acute phase, it might prolong the time to relapse.

### Family intervention

The standard family intervention for BD targets the whole family and not only the patient and includes elements of psychoeducation, communication enhancement and problem-solving skills training. It also includes support and self-care training for caregivers. Fifteen papers concerning the efficacy of family intervention in BD were found.

The first study on this intervention took part in 1991 and reported that carer-focused interventions improve the knowledge of the illness [64]. Since then, there have been a number of studies which in general support the use of adjunctive family-focused treatment. There are different designs and approaches which were tested in essentially open trials.

One intervention design consists of 21 1-h sessions which combine psychoeducation, communication skills training and problem-solving training. The sessions take place at home and included both the patient and his/her family during the post-episode period. The treatment has shown its efficacy vs. crisis management in 101 BD patients in reducing relapses (35 vs. 54%) and increasing time to relapse (53 vs. 73 weeks, respectively) [65, 66]. It was also reported to reduce hospitalization risk compared with individual treatment (12 vs. 60%) [67]. It is important that the benefits extended to the 2-year follow-up were particularly useful for depressive symptoms, in families with high expressed emotion and for the improvement of medication adherence [66]. Similar results were reported by a study of 81 BD patients and 33 family dyads, which reported that the odds ratio for hospitalization at 1-year follow-up was related with high perceived criticism (by the patients from their relatives), poor adherence and with the relatives’ lack of knowledge concerning BD (OR: 3.3; 95% CI 1.3–8.6) [68].

Adjunctive psychoeducational marital intervention in acutely ill patients was reported to have a beneficial effect concerning medication adherence and global functioning, but not for symptoms [69]. Neither adjunctive family therapy nor adjunctive multifamily group therapy improves the recovery rate from acute bipolar episodes when compared with pharmacotherapy alone [14]. These interventions could be beneficial for patients from families with high levels of impairment and could result in a reduction of both the number of depressive episodes and the time spent in depression (Cohen *d* = 0.7–1.0) [70]. In this frame, in those patients who recovered from the intake episode, multifamily group therapy was associated with the lowest hospitalization risk [71].

Another format included a 90-min duration, delivered to caregivers of euthymic BD patients; after 15-months, it was reported to have both reduced the risk of recurrence in comparison to a control group (42 vs. 66%; NNT: 4.1 with 95% CI 2.4–19.1) and also to have delayed recurrence [72]. It was particularly efficacious in the prevention of hypomanic/manic episodes and also in the reduction of the overall family burden [73]. It had been shown before that carer-focused interventions improve the knowledge of the illness [64], reduce burden [74] and also reduce the general and mental health risk of caregivers [75].

Another format of intervention included 12 sessions of group psychoeducation for the patients and their families. It has been f**o**und superior to TAU in 58 BD patients concerning the prevention of relapses, the decrease of manic symptoms and the improvement of medication adherence [76]. Finally, the comparison of family-based therapy (FBT) vs. brief psychoeducation (crisis management) in 108 patients with BD reported that the outcome depended on the existing levels of appropriate self-sacrifice [77].

Overall, the literature supports the conclusion that interventions which focus on families and caregivers exert a beneficial impact on family members, but the effect on the patients themselves is controversial. The effect includes issues ranging from subjective well-being to general health, but it is almost certain that there is a beneficial effect on issues like treatment adherence.

### Intensive psychosocial intervention

There are three papers investigating various methods of intensive psychosocial intervention. ‘Intensive’ psychotherapy has been tested on 293 acutely depressive BD outpatients in a multi-site study. Patients were randomized to 3 sessions of psychoeducation vs. up to 30 sessions of intensive psychotherapy (family-focused therapy, IPSRT or CBT). The results suggested that the intensive psychotherapy group showed higher recovery rates, shorter times to recovery and greater likelihood of being clinically well in comparison to patients on short intervention [78]. The functional outcome was also reported to be better after 1 year [79]. A second trial randomized 138 BD patients to receive collaborative care (contracting, psychoeducation, problem-solving treatment, systematic relapse prevention and monitoring of outcomes) vs. TAU. The results suggested that collaborative care had a significant and clinically relevant effect on the number of months with depressive symptoms, as well as on severity of depressive symptoms, but there was no effect on symptoms of mania or on treatment adherence [48].

### Cognitive remediation (CR) and functional remediation (FR)

Cognitive remediation and functional remediation tailored to the needs of BD patients include education on neurocognitive deficits, communication, autonomy and stress management. There are five papers on the efficacy of CR and FR.

One uncontrolled study in 15 BD patients applied a type of CR and focused on mood monitoring and residual depressive symptoms, organization, planning and time management, attention and memory. The results suggested that there was an improvement of residual depressive symptoms, executive functions and general functioning. Patients with greater neurocognitive impairment had less benefit from the intervention [80]. The combination of neurocognitive techniques with psychoeducation and problem solving within an ecological framework was tested in a multicentre trial in 239 euthymic BD patients with a moderate–severe degree of functional impairment (*N* = 77) vs. psychoeducation (*N* = 82) and vs. TAU (*N* = 80). At end point, the combined programme was superior to TAU, but not to psychoeducation alone [81, 82]. Finally, a small study in 37 BD and schizoaffective patients tested social cognition and interaction training (SCIT) as adjunctive to TAU (*N* = 21) vs. TAU alone (*N* = 16). There was no difference between groups concerning social functioning, but there was a superiority of the combination group in the improvement of emotion perception, theory of mind, hostile attribution bias and depressive symptoms [83]. A post hoc analysis using data of 53 BD-II outpatients compared FR vs. psychoeducation and vs. TAU, but the results were negative [84].

### Mindfulness-based interventions

Mindfulness-based intervention aims to enhance the ability to keep one’s attention on purpose in the present moment and non-judgmentally. Specifically for BD patients, it includes education about the illness and relapse-prevention, combination of cognitive therapy and training in mindfulness meditation to increase the awareness of the patterns of thoughts, feelings and bodily sensations and the development of a different way (non-judgementally) of relating to thoughts, feelings and bodily sensations. It also promotes the ability of the patients to choose the most skilful response to thoughts, feelings or situations. There are eight studies on the efficacy of mindfulness-based intervention in BD.

The first study concerning the application of mindfulness-based cognitive therapy (MBCT) in BD tested it vs. waiting list and included only eight patients in each group. The results suggested a beneficial effect with a reduction in anxiety and depressive symptoms [85]. A second study included 23 BD patients and 10 healthy controls and investigated MBCT vs. waiting list and the results were compared with those of 10 healthy controls. The results suggested that following MBCT, there were significant improvements in BD patients concerning mindfulness, anxiety and emotion regulation, working memory, spatial memory and verbal fluency compared to the waiting list group [86]. The biggest study so far concerning MBCT included 95 BD patients and tested MBCT as adjunctive to TAU (*N* = 48) vs. TAU alone (*N* = 47) and followed the patients for 12 months. The results showed no difference between treatment groups in terms of relapse and recurrent rates of any mood episodes. There was some beneficial effect of MBCT on anxiety symptoms [87, 88]. Recently, the focus has expanded to analyze the impact of MBCT on brain activity and cognitive functioning in BD, but the findings are difficult to interpret [86, 89, 90].

A study which applied dialectical behaviour therapy in which mindfulness represented a large component also reported some positive outcomes [91]. One study on mindfulness training reported negative results in BD patients [92].

In conclusion, the literature does not support a beneficial effect of MBCT on the core issues of BD. There are some data suggesting a beneficial effect on anxiety in BD patients. So far, there are no data supporting its efficacy in the prevention of recurrences.

## Discussion

The current review suggests that the literature supports the usefulness only of psychoeducation for the relapse prevention of mood episodes and unfortunately only in a selected subgroup of patients at an early stage of the disease who have very good if not complete remission of the acute episode. On the other hand, CBT and IPSRT could have some beneficial effect during the acute phase, but more data are needed. Mindfulness interventions could only decrease anxiety, while interventions to improve neurocognition seem to be rather ineffective. Family intervention seems to have benefits mainly for caregivers, but it is uncertain whether they have an effect on patient outcomes. A summary of the specific areas of efficacy for each of the above-mentioned interventions is shown in Table 1.

**Table 1:** Specific psychosocial interventions and their targeted therapeutic effect in BD

Intervention	Efficacy					
	Relapse/recurrence	Manic symptoms	Depressive symptoms	Anxiety	neurocognition	Overall functioning
CBT	No	–	Yes	–	–	–
Psychoeducation	Yes	No	No	–	–	Yes
IPSRT	Eq	Eq	Eq	–	–	–
Family intervention	No	No	No	–	–	No
Intensive psychosocial intervention	–	–	–	–	–	–
Cognitive remediation	No	No	No	–	No	No
Mindfulness-based interventions	No	No	No	Yes	–	–

*Eq* equivocal.

An additional important conclusion is that concerning the quality of the data available: the studies on BD patients suffer from the same limitations and methodological problems as all psychotherapy trials do. It is well known that this kind of studies suffers from problems pertaining to blindness and the nature of the control intervention. Additionally, the training of the therapist and the setting itself might play an important role. It is quite different to apply the same intervention in specialized centres than in real-world settings in everyday clinical practice. Even worse, research is not done in a standardized way and the gathering of data is far from systematic. The studies are rarely registered, adverse events are not routinely assessed, outcomes are not hierarchically stated a priori and too many post hoc analyses have been published without being stated as such. There is a lack of replication of the same treatment by different research groups under the same conditions.

There are different theories on the mechanisms responsible for the efficacy of the psychosocial treatments. One suggestion concerns the enhancement of treatment adherence [93], while another proposes that improving lifestyle and especially biological rhythms, food intake and social zeitgebers could be the key factors [60]. Also, it has been proposed that the mechanism concerns the changing of dysfunctional attitudes [23], the improvement of family interactions [94] or the enhanced ability for the early identification of signs of relapse [35].

Overall, it seems that psychosocial interventions are more efficacious when applied on patients who are at an early stage of the disease and who were euthymic when recruited [14, 95]. According to these post hoc analyses, a higher number of previous episodes [13, 40] as well as a higher psychiatric morbidity and more severe functional impairment [96] might reduce treatment response, although the data are not conclusive [97]. Also, a differential effect has been proposed with neuroprotective strategies being better during the early stages [98] and rehabilitative interventions being preferable at later stages [99].

It is unclear whether IPSRT and CBT are efficacious during the acute episodes, but there are some data in support [13, 60, 78]. Maybe specific family environment characteristics might influence the response to treatment [70, 100]. Probably, there were subpopulations who especially benefited from these treatments [13, 70], but these assumptions are based on post hoc analyses alone.

It should be mentioned that most of the research concerns pure and classic BD-I patients, although there are some rare data concerning special populations such as BD-II [36, 62], schizoaffective disorder [101, 102], patients with high suicide risk [85, 103, 104] and patients with comorbid substance abuse [105, 106].

It is interesting to note that the literature suggests that the benefits of psychosocial interventions if achieved could last for up to 5 years [36, 107], although some patients might need booster sessions [23, 108]. The complete range of the effect these interventions have is still uncharted. Although it is reasonable to expect a beneficial effect in a number of problems, including suicidality, research data on these issues are virtually non-existent [103, 104].

## Conclusions

In conclusion, the literature supports the notion that adjunctive specific psychological treatments can improve specific illness outcomes. Although the data are rare, it seems reasonable that any such intervention should be applied as early as possible and should always be tailored to the specific needs of the patient in the context of personalized patient care, since it is accepted that both the patients and their relatives have different needs and problems depending on the stage of the illness.
